# Influence of the Surface Functionalization on the Fate and Performance of Mesoporous Silica Nanoparticles

**DOI:** 10.3390/nano10050916

**Published:** 2020-05-09

**Authors:** Miguel Gisbert-Garzarán, María Vallet-Regí

**Affiliations:** 1 Departamento de Química en Ciencias Farmacéuticas, Universidad Complutense de Madrid, Instituto de Investigación Sanitaria Hospital 12 de Octubre i + 12, Plaza Ramón y Cajal s/n, 28040 Madrid, Spain; 2 Networking Research Center on Bioengineering, Biomaterials and Nanomedicine (CIBER-BBN), 28029 Madrid, Spain

**Keywords:** mesoporous silica nanoparticles, cancer, drug delivery, targeting, biological barriers, endosomal escape, stimuli-responsive, controlled drug release, nanomedicine

## Abstract

Mesoporous silica nanoparticles have been broadly applied as drug delivery systems owing to their exquisite features, such as excellent textural properties or biocompatibility. However, there are various biological barriers that prevent their proper translation into the clinic, including: (1) lack of selectivity toward tumor tissues, (2) lack of selectivity for tumoral cells and (3) endosomal sequestration of the particles upon internalization. In addition, their open porous structure may lead to premature drug release, consequently affecting healthy tissues and decreasing the efficacy of the treatment. First, this review will provide a comprehensive and systematic overview of the different approximations that have been implemented into mesoporous silica nanoparticles to overcome each of such biological barriers. Afterward, the potential premature and non-specific drug release from these mesoporous nanocarriers will be addressed by introducing the concept of stimuli-responsive gatekeepers, which endow the particles with on-demand and localized drug delivery.

## 1. Introduction

In the last few decades, the application of nanotechnology to medicine, the so-called nanomedicine, has attracted much interest among the scientific community, and it is expected to revolutionize the biotechnological and healthcare industries in the near future [1,2,3]. In this sense, the efforts of many nanotechnologists have been headed toward the development of nanoparticles for the treatment and/or diagnosis of several diseases [4,5,6]. From a general point of view, those nanoparticles can be classified as organic or inorganic. Organic nanocarriers include liposomes [7], polymeric micelles [8] or polymeric nanoparticles [9], whereas examples of inorganic nanocarriers are metal [10], carbon [11] and silica nanoparticles, which have attracted great attention owing to their excellent properties [12].

Bulk ordered mesoporous silica materials were first reported in the early 90s by researchers from Waseda University [13] and the Mobil Oil Corporation [14]. They have been employed in a number of fields, including catalysis [15,16] or energy storage [17,18], among others. In addition, these materials have been extensively applied for biomedical purposes, especially since Vallet-Regí and coworkers first reported these materials as convenient carriers for therapeutic payloads [19]. 

Because these bulk mesoporous silica materials exhibited remarkable physico-chemical properties and promising biomedical applications, their translation to the nanoscale dimension was promptly achieved. These efforts led to mesoporous silica nanoparticles (MSNs) offering (a) tunable pore size distributions and high pore volumes (2–30 nm and ca. 1 cm^3^/g, respectively), (b) high specific surface areas (up to 1500 cm^2^/g), (c) high density of silanol groups on the surface, (d) robust silica framework that allows harsh reaction conditions and (e) great biocompatibility [20,21]. 

Silica is “generally recognized as safe” by the US Food and Drug Administration (FDA) and it is often used as dietary supplement and as excipient in drug formulations [12,22]. Silica can be found as crystalline or amorphous materials, as MSNs are. Compared to the crystalline form, amorphous silica is rapidly cleared from the lungs, which would account for its lower toxicity [23]. Amorphous silica nanoparticles can be engineered into porous or nonporous silica nanoparticles. Both types of particles are hydrolytically degraded over time into water-soluble, biocompatible silicic acid, which is eventually excreted in the urine [24]. However, it should be mentioned that porous silica nanoparticles degrade faster than their nonporous counterparts, which would facilitate their excretion. This phenomenon has been ascribed to presence of mesopores and the larger surface area of the former [25]. Moreover, the degradation rate of porous silica can be tuned by functionalizing the surface with different functionalities thanks to the creation of a protective barrier [26]. Furthermore, it has been shown that MSNs can also be degraded within cells [27,28,29].

Given the suitability of MSNs to be applied for biomedical applications, much emphasis should be placed on the surface, as it constitutes the frontline of the nanocarrier. It is involved in all the interactions with the surrounding biological milieu and, consequently, it should be conveniently engineered to avoid any potential issues derived from the administration of the particles. In this regard, presenting a surface full of silanol groups is a feature of major importance, since they can be easily derivatized to other functional groups (amine, carboxylic acid, thiol, etc.) to then introduce additional molecular structures with many different functionalities.

As a result of all the excellent properties described above, MSNs have been applied for the treatment of a number of diseases, such as infection and osteoporotic scenarios [30,31], heart diseases [32,33,34], ophthalmological diseases [35,36] or diabetes [37,38], among others. In particular, most of the efforts have been headed towards the development of nanocarriers for cancer treatment, which is one of the leading causes of death worldwide [39].

**Figure 1:**
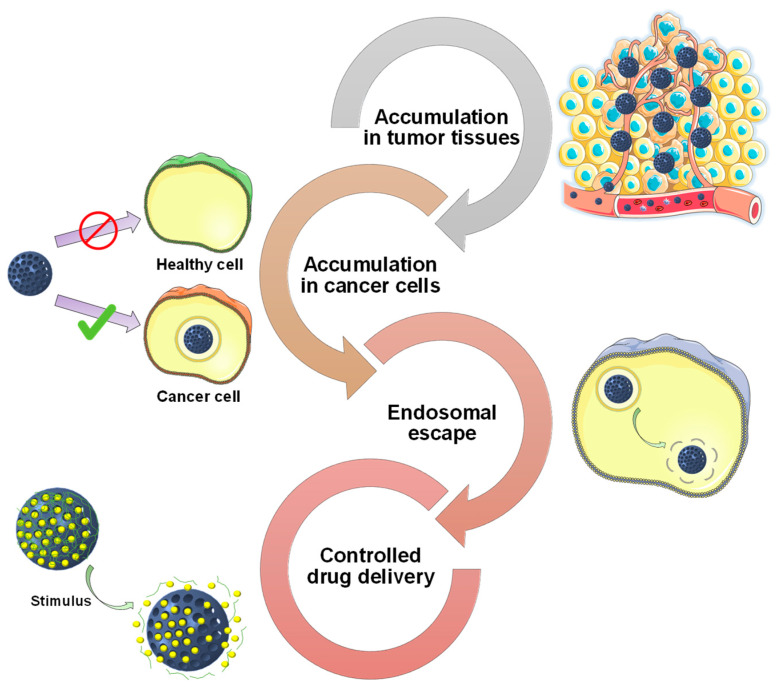
Schematic representation of relevant barriers that mesoporous silica nanoparticles (MSNs) have to face when administered to a patient. These barriers include potential premature release and different biological barriers, such as lack of accumulation in tumor tissues, lack of accumulation in cancer cells and sequestration in the endo-lysosomes.

Ideally, drug-loaded nanoparticles should accumulate only in the tumor. However, the several barriers that nanoparticles have to face when administered may not only prevent their successful translation into the clinic, but also reduce the efficacy of the treatments. In fact, a recent meta-analysis argued that less than 1% of the administered nanoparticles finally reached the tumor [40]. Figure 1 illustrates relevant barriers that nanoparticles have to face when administered to a patient.

As observed in Figure 1, MSNs should be able to: (a) accumulate preferentially in the tumors, (b) internalize selectively in cancer cells and (c) achieve endosomal escape to exert their therapeutic action. In addition, drug-loaded MSNs should be able to release their cargo only inside the tumoral cells. To obtain such behavior, researchers have focused on the development of stimuli-responsive gatekeepers. These structures are able to block the pore entrances until some specific stimulus is applied, leading to the drug release.

This review is intended to provide a description on how the surface functionalization of MSNs influences their performance as drug delivery carriers for cancer treatment. The review is sequentially organized according to the journey that MSNs would have when administered systemically to a patient. First, different strategies to enhance the accumulation of MSNs in tumoral tissues will be presented. Then, the different targeting moieties that confer the particles selective recognition of tumoral cells will be described. Afterward, various strategies for achieving the endosomal escape will be outlined. Finally, different strategies to prevent premature drug release and achieve on-demand stimuli-responsive drug delivery will be presented.

## 2. Targeting Tumor Tissues

While achieving preferential cellular uptake in cancer cells is of major importance, maximizing the amount of nanoparticles delivered to the tumoral tissues still needs to be addressed. In this sense, the use of passive and active strategies to promote the accumulation of particles constitutes a promising tool for the effective delivery of chemotherapeutics to the tumors.

### 2.1. Passive Targeting of Tumor Tissues

#### 2.1.1. Enhanced Permeability and Retention Effect

**Figure 2:**
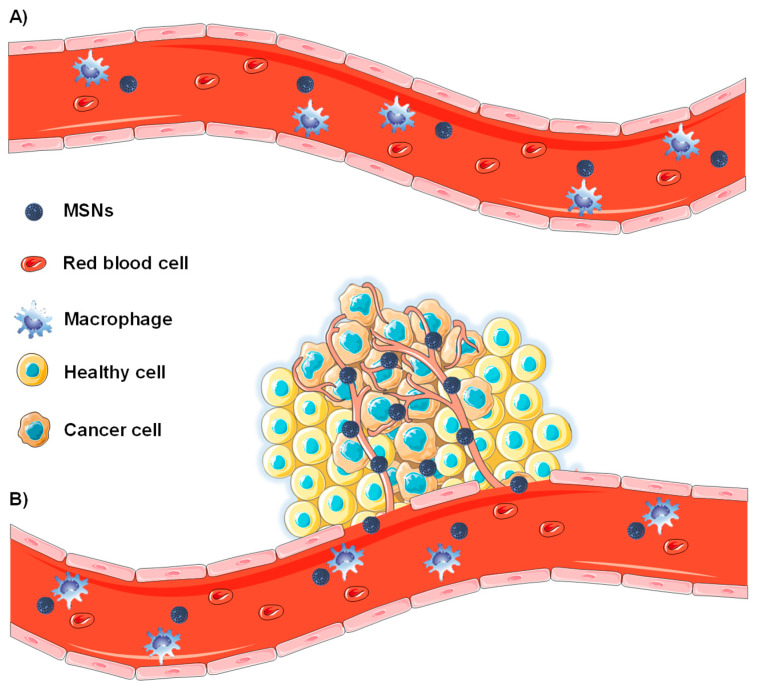
Schematic representation of the enhanced permeability and retention effect. (**A**) Normal blood vessels do not present fenestrations and MSNs remain in the bloodstream. (**B**) Tumor tissues present defective blood vessels and MSNs can leak out from them through the endothelial gap-gap and accumulate in the tumor.

The first steps towards the development nanocarriers for cancer treatment were the findings that Maeda and coworkers reported in 1986. They found that proteins above 40 kDa spontaneously accumulated in tumoral tissues and remained there for long periods of time [41]. They observed that solid tumors present impaired blood vessels, with 200–2000 nm endothelial cell-cell gaps, and poor lymphatic drainage, as a consequence of their rapid growth. As a consequence of such physiology, nanoparticles tend to leak out from the tumor vessels and accumulate in the tumors (Figure 2).

This effect is known as enhanced permeability and retention (EPR) effect and it is the basis of some commercialized nanomedicines [42]. As observed in Figure 2, only tumor blood vessels show such impaired development and, consequently, the EPR effect constitute a differential feature for the delivery of nanoparticles to tumors [43].

#### 2.1.2. Nanoparticle Features Affecting the Biodistribution of MSNs

In order for the carriers to accumulate in the tumor, it is still necessary to avoid renal clearance and removal from the bloodstream by the mononuclear phagocyte system (MPS). Regarding clearance, in vivo animal studies have shown that MSNs are mainly excreted in the urine, especially during the first two days. Furthermore, 7-nm silica nanoparticles (c-dots) have been approved by the FDA for imaging purposes and the human clinical trials have demonstrated that they are well tolerated and mainly excreted through the kidneys [24].

Features such as their size, shape or surface characteristics directly affect the final fate of the particles. With regard to the size, it is agreed that particles must be at least 10 nm in diameter to bypass renal clearance and smaller than 400 nm, so they can extravasate and diffuse into the tumor. Nonetheless, the precise size to balance those factors along with the eventual cellular uptake of the carriers is controversial. In this respect, some authors consider a size of ca. 100 nm or below as the most effective [44], while others propose a size of ca. 300 nm [45].

The biodistribution and the interaction with cells are also influenced by the shape of the carriers. Unlike their spherical counterparts, rod-like nanoparticles circulate longer in the bloodstream, being at the same time more prone to diverge closer to the vessel walls, enhancing their extravasation [46,47]. Nonetheless, it is unclear whether non-spherical particles present greater cellular internalization than their spherical counterparts [48,49].

**Figure 3:**
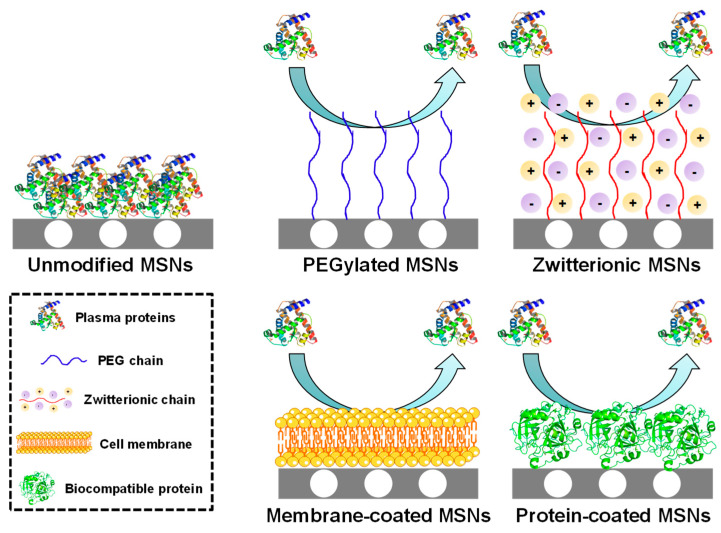
Schematic representation of the strategies employed to minimize protein adsorption and subsequent nanoparticle removal from the bloodstream. Unmodified MSNs tend to adsorb plasma protein, thereby triggering their clearance, whereas functionalized nanoparticles repel plasma protein and achieve longer circulation time.

In addition to size and shape, the surface characteristics also play an important role. There are some plasma proteins, known as opsonins, whose function is to adhere to foreign bodies so they can be easily recognized by the organism and removed from the bloodstream by the MPS. In consequence, when nanoparticles are exposed to biological fluids, these proteins are rapidly deposited onto the surface, forming a protein corona that provides a biological entity to the particles and triggers their removal [50]. That protein adsorption can be minimized if the surface is conveniently engineered (Figure 3).

A common strategy consists in the use of polyethylene glycol (PEG), which is a hydrophilic polymer that creates a hydration layer around the particles, reducing protein adsorption and improving their colloidal stability [51], as demonstrated using MSNs [52,53]. An alternative strategy involves functionalizing the surface with zwitterionic moieties (equal number of positive and negative charges). In this manner, the surface presents zero net charge, which creates a hydration layer that prevents opsonization [54], as it has been shown for different zwitterionic MSNs [55,56,57]. In addition to those purely chemical approaches, the use of red blood cell membrane coatings onto MSNs has been proved to be useful for preventing immune response and enhancing the circulation time [58]. Furthermore, coating the MSNs with cancer cell membranes not only enhances their circulation time, but also promotes the internalization in such cancer cells [59,60,61]. Finally, functionalizing MSNs with biocompatible proteins has been proved to reduce macrophage activation and minimize the immune response [62,63].

### 2.2. Active Targeting of Tumor Tissues

As mentioned above, the EPR effect constitutes a reliable approximation to the passive accumulation of particles in tumors. However, its magnitude highly depends on the particularities of the patient and the tumor [64]. For instance, it is very pronounced in Kaposi sarcoma and multiple myeloma, whereas pancreatic cancer barely exhibits EPR-mediated accumulation [65]. That non-universality of the EPR effect has fueled the development of active approaches to improve the delivery of nanoparticles to the tumor tissues. Examples of this strategy include the use of tumor-tropic peptides and tumor-tropic cells, among others.

#### 2.2.1. Tumor-Tropic Peptides

Tumor-tropic peptides are cyclic peptides that have been observed to trigger the spontaneous accumulation of MSNs in tumoral tissues at the same time that promote their penetration toward inner areas of the tumor [66,67,68]. This phenomenon is consequence of an existing endocytic transcytosis pathway in tumor endothelial cells that can be activated by such peptides. The most relevant example is the iRGD peptide, which is composed by the integrin-binding RGD (arginine-glycine-aspartate) peptidic sequence and the neuropilin-1 binding motif. The RGD sequence first binds the overexpressed αβ integrin present on the membrane of tumor endothelial cells. After that, a proteolytic cleavage exposes the neuropilin-1 motif (previously inactive), which interacts with the NRP-1 receptor and initiates a trans-tissue transport pathway [69,70]. This approach is not restricted to using the RGD motif, and it can be tuned to target alternative receptors (e.g., iNGR peptide [71]).

#### 2.2.2. Cells with Migratory Properties

On the other hand, there are some types of cells with migratory properties, including different bacteria and mesenchymal stem cells, among others [72,73,74]. For instance, there are bacteria that move toward hypoxic areas, as inner areas of tumors are [75,76]. In this sense, it has been shown that MSNs can be attached to the bacteria wall. Then, such bacteria can move toward inner areas of a 3D tumoral matrix model, carrying the particles and showing great efficacy in killing cancer cells [77].

Mesenchymal cells show inherent migratory properties in response to injury or inflammation and their main advantage is their low or non-immune response. In this manner, nanoparticles are first internalized within these cells to then be spontaneously delivered to the tumoral tissues without exposing them to the biological milieu, thereby minimizing any potential immune response against the particles, as demonstrated using MSNs [78,79,80].

### 2.3. Enhanced Penetration in the Tumoral Mass

Aside from targeting the tumoral mass, nanoparticles should diffuse toward inner areas of the tumor to reach and eliminate all cancer cells. The extracellular matrix is predominantly composed of a highly interconnected network of collagen and other components such as hyaluronic acid (HA), elastin, laminin and proteoglycans. Besides, tumors present elevated interstitial pressure [81,82]. As a result, nanoparticles penetration and diffusion are hindered, thereby decreasing the efficacy of the treatment.

#### 2.3.1. Proteolytic Enzymes

Some authors have proposed the use of proteolytic enzymes, as they are able to digest the components of the extracellular matrix, therefore decreasing its stiffness and enhancing nanoparticle penetration. For instance, the surface modification of MSNs with bromelain enhances their diffusion in the tumoral mass, compared to the uncoated particles [83]. However, this strategy has some limitations, as enzymes may degrade and lose their catalytic activity on their way to the tumor. In this regard, our group recently reported the encapsulation of collagenase within a degradable polymeric mesh. This coating remained unaffected at physiological pH, preserving the catalytic activity of the enzyme. However, the capsule degraded at acid pH, releasing the enzyme and enhancing the penetration of MSNs in a 3D tumoral matrix model thanks to the collagen digestion [84,85].

It is agreed that smaller nanoparticles penetrate deeper in the tumors, although those with larger size present greater circulation time. For that reason, a nice strategy to address both concerns consists in designing nanoparticles able to undergo a larger-to-smaller size change upon arrival to the tumoral mass [86]. In this regard, a recent article proposed the synthesis of small 40 nm MSNs that were then engineered as large nanocarriers through the use of 3-arm PEG. MSNs were connected using a peptidic sequence (GPLGIAGQ) cleavable by metalloproteinases (MMPs), which are overexpressed in the extracellular tumoral matrix. Hence, the particles only reduced their size once in the tumor, enhancing the initial accumulation and subsequent penetration in the tumor [87].

#### 2.3.2. Ultrasounds

An alternative strategy to enhance the penetration of particles in tumors consists in the use of localized ultrasound (US) [88]. The rationale for using US relies on the inertial cavitation phenomenon. Cavitation is the oscillation of gas bubbles in a fluid, which can be stable (expanding and contracting around a given radius) or instable if the pressure is high enough (inertial cavitation). In the latter scenario, the bubbles grow unstably and collapse violently during compression, effect that can be taken advantage of for impelling the particles, favoring their extravasation and subsequent penetration in the tumoral mass [89]. It this sense, it has been observed that applying US leads to increased tumor vascular disruption and deeper particle penetration [90]. However, our group has demonstrated that too high pressure might be needed to obtain significant penetration. In this regard, it has been shown that co-administering MSNs with submicrometric cavitation nuclei leads to enhanced diffusion of the particles in an agarose model upon application of clinically suitable US frequency [91].

## 3. Targeting Cancer Cells

As stated above, the shape and size of nanocarriers not only determine their extravasation but also affects their interaction with cells. In addition to these parameters, the surface charge also plays an important role. Because of the negatively charged cell membrane, cationic nanoparticles show greater uptake, albeit being more prone to undergo opsonization and subsequent clearance [92,93]. However, there is evidence that positive nanoparticles tend to accumulate in the periphery of the tumor whereas those negatively charged tend to penetrate deeper owing to the repulsion with cell membranes [94]. Nonetheless, even if the previously mentioned factors were optimized, nanoparticles might still be internalized by healthy cells. For that reason, there has been much research on how to specifically recognize cancer cells as well as on how to optimize intracellular trafficking.

**Figure 4:**
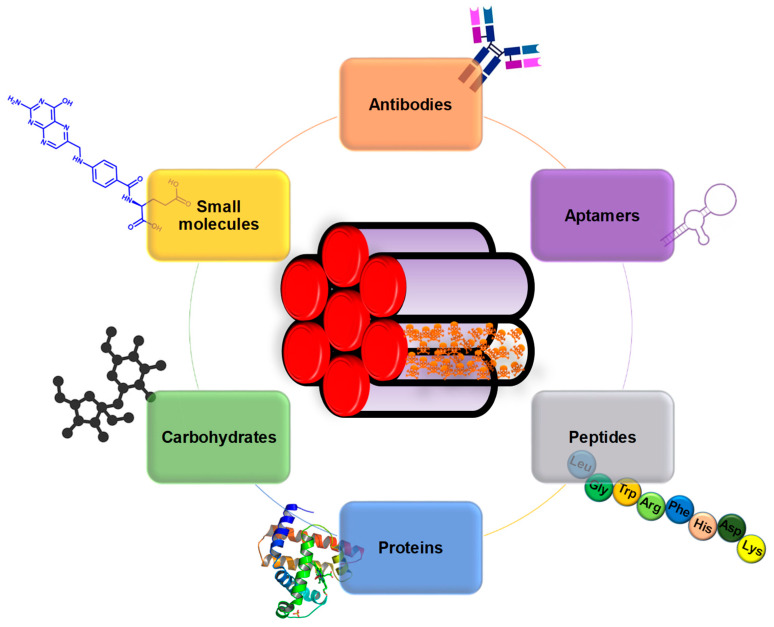
Schematic representation of targeting ligands employed for MSNs-based targeted drug delivery.

A widely employed strategy for the selective targeting of cancer cells consists in the functionalization of the surface targeting ligands that are able to bind specific receptors that are overexpressed only on the membrane of tumoral cells. The ligand density on the particles is a parameter of key importance. For instance, too high ligand density would account for (a) reduced particle stealth character (i.e., increased clearance), (b) increased particle size (i.e., reduced accumulation in the tumor via EPR effect), (c) steric hindrance of closely packed ligands (i.e., reduced nanoparticle binding ability) and (d) high number of cells receptors used per particle (i.e., reduced cellular uptake) [95]. Examples of such targeting agents include antibodies, aptamers, peptides, proteins, saccharides and small molecules (Figure 4).

**Figure 5:**
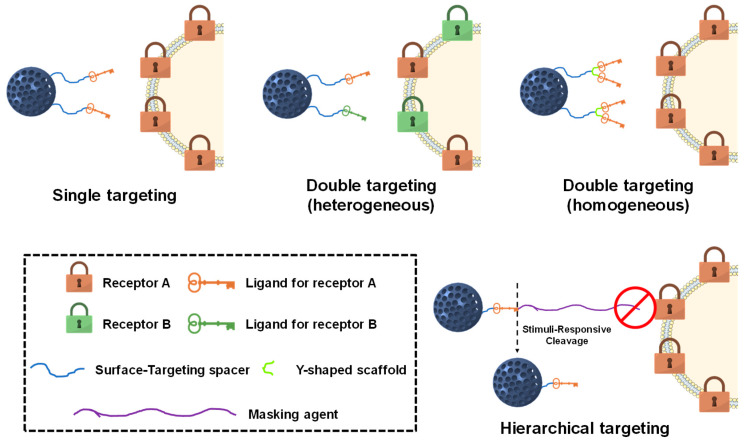
Schematic representation of the most common targeting approaches using MSNs.

Such targeting agents can be implemented into MSNs following different configurations, endowing the particles with many different targeting possibilities. A schematic representation of the strategies most commonly employed to target cancer cells with MSNs is shown in Figure 5.

### 3.1. Single Targeting

The simplest approximation involves the direct attachment of those targeting ligands to the surface of MSNs. In this manner, the targeting ligands are permanently exposed and can interact with the surrounding environment.

#### 3.1.1. Antibodies

Antibodies bind specific antigens located on the membrane of cancer cells with extremely high specificity. For instance, trastuzumab, an FDA-approved monoclonal antibody that targets the HER2 receptor, has been extensively conjugated with MSNs, endowing them with remarkable targeting ability toward SK-BR3 [96,97,98] and BT-474 [98,99,100] breast cancer cells. Similarly, the FDA has also approved a monoclonal antibody for targeting the CD44 receptor, whose grafting to MSNs results in enhanced cellular uptake in MCF-7 breast cancer cells [101].

TRC105, a monoclonal antibody in clinical trials that binds the CD105 membrane protein, has also proved to be useful for improving the internalization of MSNs 4T1 breast cancer cells [102,103,104]. A monoclonal antibody for targeting the EpCAM transmembrane protein has also been FDA-approved, and it has been grafted to MSNs to enhance their efficacy against Y79 retinoblastoma cells [105]. Another monoclonal antibody that is currently in clinical trials is TAB-004. This compound targets the MUC1 transmembrane glycoprotein, which is overexpressed in most of cancer cells, and its conjugation with MSNs has yielded excellent results targeting murine breast cancer cells expressing the human form of MUC1 [106].

Finally, cetuximab, which is a monoclonal antibody also approved by the FDA, has been grafted to MSNs to selectively target overexpressed EGFR receptors in MCF-7 breast cancer [107], PC9 non-small lung cancer [108] and in a number of pancreatic cancer cell lines [109]. Even though they are highly effective in binding such receptors, their high production cost and potential undesired immune response have fueled the research of alternative targeting agents.

#### 3.1.2. Aptamers

Aptamers are single-stranded DNA, RNA or unnatural oligonucleotides able to adopt tertiary conformations that exhibit affinity for various types of targets. These structures show specificity comparable to that of antibodies, besides being non-immunogenic and easy to synthesize [110]. For instance, the EpCAM protein can also be targeted using conveniently engineered aptamers. In this regard, modifying the surface of MSNs with such aptamer increases their selectivity toward Huh-7 liver cancer cells [111], HepG2 hepatic cancer cells [112] and different colon cancer cells [113,114]. Similarly, the MUC1 protein can also be targeted using aptamers, increasing the ability of MSNs to inhibit the proliferation of MDA-MB-231 [115] and MCF-7 breast cancer cells [116]. The AS1411 aptamer was the first aptamer to enter clinical trials, and it has been proved to be effective in targeting the nucleolin receptor of HeLa [117], SKOV-3 ovarian cancer cells [118] and MCF-7 breast cancer cells [119,120,121] with MSNs. Additional examples of aptamer-targeted MSNs are those using the YQ26 or HB5 aptamers, which show high selectivity for END-positive [122] and HER2-positive [123] cancer cells, respectively.

#### 3.1.3. Peptides

The use of cell-penetrating peptides has attracted much attention owing to their ability to cross biological membranes [124]. The exact mechanism remains not fully understood but there are some proposed mechanisms [125]. For instance, the functionalization of MSNs with the TAT peptide provides great cellular uptake and further targets the nanoparticles to the cell nucleus [126]. Another example is the functionalization of MSNs with the KALA peptide, which is able to mediate the internalization and subsequent endosomal escape of nanoparticles [127].

The examples described so far rely on nonspecific internalization mechanisms, meaning that those particles could be internalized by both tumoral and healthy cells. For that reason, researchers have focused on identifying specific peptidic sequences that provide specific binding of overexpressed cellular receptors. For instance, functionalizing the surface with the RGD peptide is useful for promoting the internalization of MSNs in cells overexpressing αβ-integrin, such as many cancer cells [128,129,130,131,132] or the tumor endothelium [133]. The CD13 receptor, which is upregulated in glioma cells, can be efficiently targeted using NGR-functionalized MSNs [134,135]. Moreover, MSNs bearing this peptide are more prone target brain endothelial cells and cross the blood-brain-barrier [136]. In addition to those wide spectrum peptides, MSNs can be functionalized with peptides that show affinity for receptors present only on very specific cell lines. Examples of these peptides include NAPamide (targets the melanocortin-1 receptor of melanoma cells) [137], Bld-1 (targets formyl peptide receptor-1 of bladder cancer cells) [138] or IL-13 (targets the IL-13R-α2 receptor of glioma cells) [139].

#### 3.1.4. Proteins

Proteins also have a relevant role in targeting cancer cells. For instance, transferrin is a protein that mediates iron cellular uptake and its receptor is highly overexpressed in many cancer cells. In this sense, transferrin-targeted MSNs show enhanced cellular uptake in HT1080 fibrosarcoma [140], HepG2 hepatocellular carcinoma [141], Huh-7 liver cancer cells [142], MDA-MB-231 breast cancer cells [143], C6 glioma cells [144] and MIA PaCa-2 pancreatic cancer cells [145]. A unique feature of pancreatic cancer cells is the presence of upregulated urokinase plasminogen activator receptors, which can be efficiently targeted by modifying the surface of MSNs with the serine protease urokinase plasminogen activator [146]. Lectins show affinity for aberrantly overexpressed carbohydrates on the membrane of cancer cells. In this regard, concanavalin A-coated MSNs present superior internalization in cells overexpressing sialic acid residues [147], whereas *Aleuria aurantia* promotes the internalization of MSNs in colon adenocarcinoma cells upregulating the sialyl-Lewis X antigen [148].

#### 3.1.5. Carbohydrates

These biomolecules have also been explored as targeting agents owing to their ability to interact in a very specific manner with overexpressed lectin proteins present on the cell membrane. For instance, the asialoglycoprotein receptor can be targeted using MSNs functionalized with galactose [149] or lactobionic acid [150,151,152,153]. In addition, the conjugation of glucose derivatives with MSNs are useful for targeting overexpressed GLUT receptors [154,155]. The CD44 receptor can also be targeted using carbohydrates, and it has been shown that modifying the surface of MSNs with HA [156,157,158,159] or chondroitin sulfate [160,161,162] promotes the cellular uptake in CD44-positive cancer cells.

#### 3.1.6. Small Molecules

There are also some small molecules that are commercially available or easy to synthesize that can be employed to target overexpressed cellular receptors. For instance, cancer cells need huge amounts of vitamins as a consequence of their accelerated metabolism and many of their receptors are highly overexpressed [163]. Examples are the use of folic acid (vitamin B9) [164,165,166,167,168,169,170,171,172] and biotin (vitamin B7) [173,174,175], whose conjugation with nanoparticles enhances their cellular uptake in a number of cancer cell lines. Additionally, it seems that cobalamin (vitamin B12) could have a role in enhancing cellular uptake [176].

Commercially available boronic acids bind sialic acid residues [177]. For that reason, they can be employed to target MSNs to HepG2 cells showing aberrant overexpression of such residues [178]. Besides, boronic acid-functionalized MSNs are useful for detecting the presence of glycosylated proteins, which have a role in the initiation and progression of tumors [179]. In addition, highly specific cancer cell targeting can be accomplished by synthesizing small molecules with great affinity for a very specific receptor. For instance, the norepinephrine transporter is highly overexpressed in neuroblastoma cells and can be efficiently targeted using MSNs containing benzylguanidine analogues [180]. A summary of all the above-mentioned approximations is shown in Table 1.

### 3.2. Dual Targeting

The previously mentioned active targeting agents can be combined in a single carrier so that the final nanoparticle presents exceptional targeting capacities for various cellular receptors. Nanoparticles synthesized following this philosophy are referred to as dual-targeted nanocarriers. In this manner, MSNs can be engineered so they show selectivity not only for tumoral cells but also for the tumor vasculature or certain subcellular compartments.

#### 3.2.1. Membrane Dual Targeting

The first strategy consists in using only membrane targeting agents. For instance, the combined use of HA and the RGD peptide has been shown to promote the cellular uptake of MSNs in ovarian cancer thanks to the simultaneous targeting of CD44 and αβ-integrin [181,182]. Similarly, targeting HeLa cells using both biotin and folic acid results in slightly better internalization rates than each one alone [183]. Besides, grafting the monoclonal antibody bevacizumab to MSNs bearing the EpCAM aptamer provides superior cellular uptake compared to the group containing only the aptamer [184]. However, our group recently demonstrated that using heterogeneous double targeting moieties not always provides the best results. In this case, a benzylguanidine analog with high affinity for the norepinephrine receptor of neuroblastoma was implemented in a Y-shaped, flexible scaffold, demonstrating extraordinary targeting capacity compared to any other combination [185].

#### 3.2.2. Sequential Dual Targeting

Another approach involves the use of targeting agents showing not only selectivity for cancer cells but also for other parts of the tumor. For instance, the co-conjugation of both RGD and TAT peptides on MSNs leads to sequential vascular-membrane-organelle targeting. In this manner, nanoparticles first bind the tumor vasculature to then be internalized by the tumoral cells owing to the cooperative behavior of both targeting agents. Once inside the tumoral cell, TAT drives the MSNs into the nucleus to achieve great cytotoxicity [186]. Another strategy to accumulate the particles in the nucleus consists in modifying the surface of MSNs with folic acid and dexamethasone. In this manner, folic acid first triggers the cellular uptake of the particles, which then undergo nuclear translocation thanks to the dexamethasone molecules [187]. Mitochondria can also be targeted using small molecules. For instance, MSNs can be engineered to first target the CD44 receptor using HA, which would then degrade in the lysosomes, exposing a triphenylphosphonium (TPP) derivative with strong mitochondrial affinity [188].

#### 3.2.3. Janus Dual Targeting

In addition to the previous approaches, in which the targeting agents were randomly distributed along the surface, the use of Janus nanoparticles (nanoparticles that present two hemispheres, each one with different reactivity) provides further possibilities. For instance, it is possible to decorate one hemisphere with HA to target CD44 and the other one with masked positive charges that are only exposed at the acid pH of the tumoral matrix, improving the internalization in A549 lung cancer cells [189]. Besides, our group recently reported Janus MSNs with one hemisphere bearing folic acid and the other one presenting a TPP derivative, demonstrating superior cell internalization and subsequent mitochondrial targeting [190].

### 3.3. Hierarchical Targeting

As stated in Section 2.1, nanoparticles are usually modified with PEG to endow them with stealth properties. However, the targeting moieties are usually attached to the end of the PEG chain in many of the available systems. This strategy entails two main issues. First, despite the fact that normal cells do not show overexpressed levels of the previously mentioned receptors, they still present some of them. In consequence, nanoparticles bearing the targeting moieties anchored to the PEG chains might lead to non-specific targeting of healthy tissues. Second, because those targeting agents are directly exposed to the surrounding biological milieu, they might decrease the stealth character of the particles, consequently triggering their clearance.

Considering the facts exposed above, some researchers have explored the use of hierarchically-targeted nanocarriers, where the targeting agents are masked by PEG chains. In this manner, the stealth character of the nanoparticles is preserved on their way to the tumor. In addition, PEG chains difficult the cellular internalization of nanoparticles, so off-target is minimized [191]. Finally, PEG chains are detached upon arrival to the tumoral mass, thereby exposing the targeting agents and triggering the drug release.

#### 3.3.1. pH-Responsive Hierarchical Targeting

The most extended approximation is based on using benzoic imine bonds, which are known to be hydrolyzed at the slightly acidic pH of the tumoral matrix (pH 6.4–6.8) [192]. A common approach consists in forming acid-labile benzoic imine bonds between MSNs presenting free NH_2_ groups and an aldehyde PEG. In this way, the positively charged groups are exposed only in the tumor, triggering the cellular uptake through electrostatic interactions [193,194,195]. This method can also be employed to mask the RGD peptide, combining stealth properties and subsequent pH-mediated active targeting [196]. Similarly, positive charges can be generated using a thermally-cleavable bond between PEG and the amino groups on the surface [197].

#### 3.3.2. Enzyme-Responsive Hierarchical Targeting

As stated in Section 2.3, levels of MMPs are upregulated in the tumoral matrix and can be employed for cleaving specific peptidic sequences. For instance, MSNs can be decorated with HA and further functionalized a MMP-sensitive PEGylated gelatin. In this way, the particles would accumulate in the tumor and then MMPs would cleave the PEGylated gelatin, exposing the targeting to CD44 to trigger the cellular uptake [198]. A similar strategy can be employed to engineer hierarchical folic acid-targeted MSNs [199]. The RGD peptide can also be masked using a MMP-sensitive peptidic sequence, which would be cleaved along with the stealthy coating only in the tumor tissue, thereby triggering the specific binding of αβ-integrin [200,201].

The different strategies for achieving dual and hierarchical targeting described in Section 3.2 and Section 3.3 are summarized in Table 2.

## 4. Achieving Endosomal Escape

**Figure 6:**
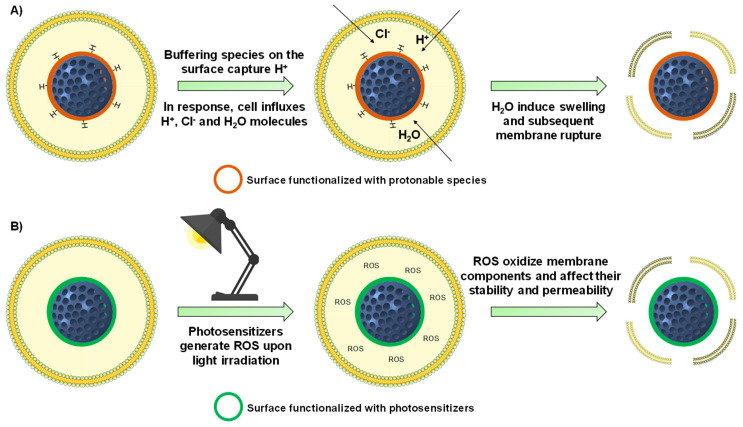
Schematic representation of the most employed strategies employed to induce the endosomal escape of MSNs. (**A**) The protonable species on the surface of the particles capture the protons of the vesicle. To counteract that basification, cell influxes protons, chloride ions and water, which induces the swelling and eventual endo-lysosomal rupture. (**B**) MSNs functionalized with photosensitizers are able to generate reactive oxygen species (ROS) upon light irradiation. These ROS can oxidize the lipid membrane of the endo-lysosomes, leading to a loss of stability and enhancement of the permeability of the lipid bilayer, triggering nanoparticle escape.

In addition to being preferentially internalized, nanoparticles should be able to release their payload properly in the cytoplasm. However, nanoparticles that are taken up through the endocytic pathway may be sequestered in the acid endo-lysosomes, which might degrade nanoparticles payload. Besides, membrane impermeable and/or poorly membrane permeable therapeutics need to be properly released into the cytoplasm to exert their action. In this sense, the development of nanoparticles with ability to achieve endosomal escape has received much attention (Figure 6) [202].

### 4.1. Internally-Triggered Endosomal Escape

#### 4.1.1. Proton Sponge Effect

The first approach consists in taking advantage of the acidic environment found in the endocytic pathway (pH 4.5–6.5) [203]. MSNs can be functionalized using macromolecules that show buffering capacity at that acidic pH, which would lead to the disruption of the endosomes/lysosomes via the “proton sponge effect”. This membrane rupture would be consequence of the cell influxing protons along with chloride ions and water to counteract the capture of protons by the particles. In this manner, the water molecules would make the endo-lysosomes to swell, eventually leading the release of the particles into the cytoplasm [202].

For instance, coating the surface of MSNs with poly(amidoamine) dendrimers results in the endosomal escape of the particles, owing to their great buffering capacity [204]. A similar example consists in the use of polyethyleneimine (PEI), a macromolecule with a huge number of protonable amino groups that can mediate the endosomal escape of MSNs [205,206]. However, it should be mentioned that it remains unclear whether PEI-mediated escape is actually consequence of its buffering capacity [207]. Because PEI-coated MSNs are positively charged, PEI can be employed to first load negatively charged nucleic acids within the polymeric mess and then induce the endosomal escape for effective therapeutic action. Examples of nucleic acids transfected using this approach include small interfering RNA (siRNA) [208,209,210] and short hairpin RNA (shRNA) [211]. In addition, it is known that PEI can be cytotoxic, depending on their molecular weight and conformation (linear, branched) [212]. For that purpose, PEI-coated MSNs can be further coated with poly(methyl vinyl ether-co-maleic acid) to yield more biocompatible nanoparticles with endosomal escape capabilities [213].

Amino acids and peptides also find application in the field of endosomal escape. For instance histidine, whose imidazole ring has a pKa of ca. 6 [214], shows buffering capacity at the pH of the endocytic pathway. For instance, it can be polymerized to engineer a pH-responsive gatekeeper for MSNs with potential endosomal escape capabilities [215]. Similarly, the surface of the particles can be modified with only the imidazole motif, which allows the effective delivery of plasmids into the cytoplasm [216]. Additionally, histidine can be incorporated within other sequences of amino acids. For instance, histidine-rich fusogenic peptides promote the destabilization of the vesicle membrane by both the proton sponge effect and fusion events with the peptide [217,218]. In addition, histidine motifs can be engineered with RGD targeting peptides to achieve both membrane targeting and endosomal escape of MSNs [219].

#### 4.1.2. Other Mechanisms for Destabilizing the Endo-Lysosomal Membrane

As stated in Section 4, cell-penetrating peptides are able to cross biological membranes. For that reason, the KALA peptide can also be employed as a means of crossing the endo-lysosomal membrane [127]. Besides, it is possible to mask their positive charge so the surface of MSNs is negatively charged in the bloodstream and clearance is minimized. However, at the acid pH of the endo-lysosomes the positive charges are recovered, triggering the endosomal escape. Examples include the use of masked polylysine [220] and TAT peptide [221]. Another approach involves the use of MSNs functionalized with lysine-containing α-helical peptides, which are able to act at the same time as gatekeepers and endosomolytic agents [222]. Finally, the escape of MSNs can also be accomplished by functionalizing the surface with TPP derivatives, which are highly cationic and lipophilic small molecules able to destabilize the endo-lysosomal membrane. [190,223,224].

### 4.2. Externally-Triggered Endosomal Escape

A second strategy for inducing endosomal escape consists in grafting macrocycles (porphyrins and phthalocyanines) to the surface of the particles to then irradiate the MSNs with a given wavelength. When these materials are exposed to light, those macromolecules generate reactive oxygen species (ROS), which are known to destabilize the membrane of the endo-lysosomes [225]. Examples of photosensitizers are porphyrins and phthalocyanines. The porphyrin PpIX generates ROS upon 405 nm ultraviolet light irradiation, and it has been proved to mediate the endosomal escape of MSNs coated with biocompatible supported lipid bilayers [226,227]. Porphyrins can also generate ROS upon visible light application [228]. Similarly, the phthalocyanine AsPCs_2a_ generate ROS upon irradiation with 639 nm near-infrared light, and its grafting to MSNs has been shown to be effective in triggering their endosomal escape [229,230]. Finally, indocyanine green can be loaded within the mesopores of MSNs and generate ROS upon 780 nm light excitation, leading to the endosomal escape of the particles [231].

## 5. Functional Groups Determine Drug Loading and Release

The outstanding textural properties of MSNs allow the loading of large amounts of therapeutics, process easily accomplished thanks to their open porous structure. However, for the very same reason, the loaded molecules might prematurely diffuse out of the pores and affect healthy tissues. Both the loading and release kinetics are governed by the interactions between such molecules and the silanol groups of the particles. Hence, tuning the functional groups present in the particles provides a first manner to improve drug loading and control premature release. In consequence, mesoporous silica matrices should be conveniently modified according to the drug to be stored.

Overall, the loading process of polar drugs can be improved using polar functional groups. Conversely, the loading of hydrophobic compounds increases in the presence of nonpolar moieties [232]. For instance, NH_2_-functionalized mesoporous silica materials provide considerably higher loading and more sustained release of alendronate [233,234]. Similar behavior is found for erythromycin when the particles are functionalized with long alkyl chains [235] and ipriflavone in the presence of phenyl groups [236], thanks to the appearance of hydrophobic interactions.

With regard to antitumoral drugs, introduction of SH groups increases the storage of cisplatin [237] and mitoxantrone [238]. Doxorubicin loading in MSNs is enhanced after functionalization with COOH or PO_3_^−^ groups, although that of paclitaxel is not improved after modifying the particles with phenyl groups [239]. Moreover, functionalization with NH_2_ or CN groups provides the greatest loading of 5-fluorouracil in MSNs [240]. In addition, the amount of gemcitabine loaded increases if MSNs are modified with a carboxylic acid derivative of piperazine [241].

Periodic mesoporous organosilica nanoparticles (PMONs), which are composed of bridged organoalkoxysilanes, offer increased loading capacity of antitumoral drugs owing to their higher hydrophobicity and isoelectric point. For instance, PMONs containing porphyrin-ethylene bridged moieties provide extraordinary loading of gemcitabine [242]. Similarly, the use of precursors bearing ethylene-bis(propyl)disulfide [243] or oxamide-phenylene moieties [244] yields PMONs with remarkable DOX loading capacity.

## 6. Stimuli-Responsive Drug Delivery

**Figure 7:**
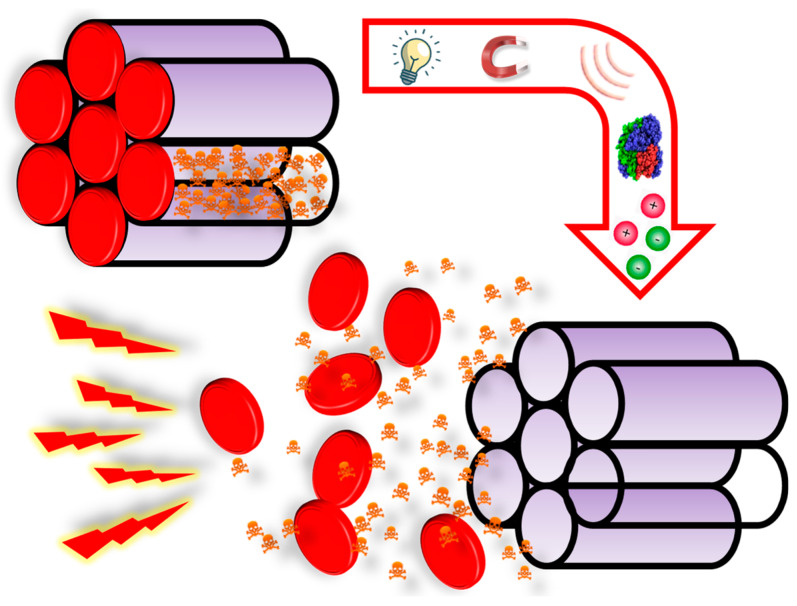
Schematic representation of how stimuli-responsive mesoporous materials work. The gatekeepers close the pore entrances and avoid premature release until some specific stimulus is applied. The stimuli can be applied from inside (e.g., pH, redox species, enzymes) or outside the patient (e.g., light, US, magnetic fields).

Modifying the interactions between the silica matrix and the guest molecules provides a first manner to diminish premature release. A step head involves the use of stimuli-responsive gatekeepers, which are structures able to open the mesopores on-demand in response to the application of a particular stimulus (Figure 7).

As shown in Figure 7, the origin of stimuli can internal or external. The use of internal stimuli relies on some relevant biological markers being upregulated/downregulated in tumor tissues (e.g., pH, redox species, enzymes). In this manner, the pore entrances of the particles would remain closed under physiological conditions, whereas the tumor microenvironment (inside or outside cancer cells) would trigger the drug release. On the other hand, external stimuli-responsive MSNs only allow drug release when the stimulus is applied from the outside using specific equipment (e.g., light, US, magnetic fields).

### 6.1. pH-Responsive MSNs

A unique feature of tumor tissues is the slightly acidic pH of the tumoral matrix, which has been quantified to be within the range 6.4–6.8 [192]. This subtle acidification is consequence of a process known as Warburg effect, which states that most cancer cells (or any proliferating cell) produce energy through the aerobic glycolysis process regardless of the presence of oxygen, leading to the secretion of large amounts of acidic lactate [245]. Such acidification plays a major role in cancer progression because (a) it promotes cancer cell migration ad radioresistance, (b) it disturbs the metabolism and function of T-cells and (c) it provokes chronic inflammation in tumor tissues by enhancing interleukin production by macrophages and T-cells [246].

In addition, there are some intracellular compartments and organelles that present slight differences in pH with respect to that of the cytoplasm, which is nearly neutral. In this sense, nanoparticles can be internalized in cells through the endocytic pathway, which results in the formation of vesicles containing the particles. These vesicles, whose function is degrading compounds no longer useful for the cells, are acidic in nature and evolve from the endocytic vesicles (pH 6.5) to the lysosomes (pH 4.5–5) [247].

Such differences in pH can be employed to trigger the drug release from pH-responsive mesoporous nanoparticles. The most common strategies to design such systems involve the use of: (1) acid-labile bonds, (2) pH-degradable gatekeepers, (3) pH-operated nanovalves, (4) polymers that undergo conformational changes upon variations in pH, (5) biomolecules that vary their charge or conformation upon changes in pH and (6) polyelectrolytes.

#### 6.1.1. Acid-Labile Bonds

The simplest approximation consists in grafting different types of gatekeepers through acid-labile bonds. These linkers are stable at physiological pH but rapidly hydrolyze when pH drops. For instance, hydrazone bonds are cleaved at pH 5 and can be employed to close the mesopores with gold nanoparticles [248,249], HA [250] or charge-reversal polymers [251]. Acetal bonds hydrolyze at pH 5 as well and find application as acid-responsive linkers between MSNs and small metal nanoparticles [252,253], graphene quantum dots (QDs) [254], polymeric coatings [255,256] and proteins [147]. Boronate esters are also interesting because they are known to undergo reversible hydrolysis at acid pH [257] and can be employed to design MSNs with on-off release behavior using small gold [258] and Fe_3_O_4_ nanoparticles [259], ZnS nanocrystals [260] or lactobionic acid [261] as gatekeepers. Imine bond-based compounds find application as crosslinking agents and allow the formation of acid pH-responsive protective layers that disassemble when pH drops. Examples are the combination chitosan with dialdehyde starch [262], dextrin with tetraethylenepentamine [263] or glutaraldehyde with polyethyleneimine [264].

#### 6.1.2. pH-Degradable Gatekeepers

Another approach is based on the use of acid-degradable gatekeepers that close the mesopores at physiological pH and degrade in the acidic subcellular compartments, triggering the drug release. For instance, small degradable nanoparticles can be employed as pore blockers. Examples include ZnO QD, whose degradation generates cytotoxic Zn^2+^ ions [265,266,267,268] and MnO nanoparticles that generate manganese ions upon dissolution that can be used for imaging [269,270]. In addition, MSNs can be coated with a layer of MgAl-hydrotalcite, which is known to degrade at acid pH [271].

There are various examples of organic gatekeepers that decompose upon pH variations as well. For instance, MSNs can be functionalized with a polydopamine layer that remains stable at physiological pH but degrade when pH drops [272]. Another approximation consists in functionalizing the particles with self-immolative moieties, which are molecules or macromolecules that present a cleavable trigger that initiate the self-degradation of the structure upon application of a very specific stimulus [273]. Such self-immolative structures endow MSNs with responsiveness to either basic [274] or acid pH [275]. In fact, the latter have been recently implemented into pH-responsive mesoporous carbon nanoparticles, validating in vivo the pH-responsiveness of such self-immolative polyurethanes [276].

#### 6.1.3. pH-Operated Nanovalves

There are some examples of MSNs gated using the so-called nanovalves, which are supramolecular gatekeepers able to close the pores upon interaction with a stalk grafted on the surface. A first strategy involves the use of triazine derivatives as stalks and 5-fluorouracil-based compounds as caps, which are able to close the pore entrances thanks to hydrogen bonding interactions. However, at acid pH the interaction weakens and cytotoxic 5-fluorouracil is released along with a loaded cytotoxic, leading to high cytotoxicity [277]. Another strategy involves the use of biocompatible cyclodextrins (CD). They present a hydrophilic outer surface and a hydrophobic cavity with which the stalk interacts. The interaction is stable at physiological pH and the CD tightly close the pore entrances. However, it weakens at acid pH, triggering the drug release. Examples of stalks employed to accomplish pH-mediated drug delivery are amine-based stalks [278,279,280] and complementary base pairs [281].

#### 6.1.4. Conformation-Changing Polymers

This kind of polymers are collapsed on the surface at a given pH, blocking the pore entrances. However, upon a variation in pH the ionizable groups of the polymer acquire net charge and repulsion forces among the chains appear. Hence, the polymeric layer changes its hydrophobicity and adopt a more extended conformation that permits the drug release. Cationic polymers showing this behavior protonate at acid pH and can be employed to design gates that open only in the acidic endo-lysosomes. Examples include amino-based acrylates and methacrylates [282,283], polyamine-based polymers/dendrimers [284,285,286] and poly(*n*-vinylpyridine) [230,287,288]. On the other hand, anionic polymers, such as poly(acrylic acid) and poly(methacrylic acid), protonate at neutral pH, closing the pores at acid pH and being excellent candidates for oral drug delivery [289,290,291].

#### 6.1.5. pH-Responsive Biomolecules

Likewise, some biomolecules undergo reversible changes upon variations in pH. For instance, MSNs can be functionalized with polypeptides containing protonable groups that would only allow drug release at a given pH. In this sense, poly(L-histidine) [215] protonates at acid pH, whereas poly(L-aspartic acid) [292] and succinylated poly(ε-lysine) [293] protonate at physiological pH and might be useful for oral drug delivery. Additionally, such pH variations can reversibly modify the 3D structure of peptides, proteins and DNA strands to allow the drug release only at acid [294,295,296,297] or physiological pH [298,299].

#### 6.1.6. Polyelectrolytes

Another approach consists in the use of polyelectrolytes, which are polymers bearing ionizable cationic or anionic groups. Such polyelectrolytes can be directly deposited on the surface, forming a single polymeric layer that interacts with the nanoparticle surface. In this manner, variations in pH would lead to electrostatic repulsions that would weaken the interaction with the surface, opening the pores. The surface must be accordingly functionalized, i.e., positively charged for anionic and negatively charged for cationic polymers. Examples include coating the surface with PEI, poly(2-diethylamino ethyl methacrylate) [300], poly(vinyl pyridine) [301], polyanionic poly(acrylic acid-co-itaconic acid) [302] and chitosan, which is particularly interesting because it swells reversibly at acid pH, leading to on-off systems [303,304,305,306]. Furthermore, polyelectrolytes can be disposed forming multilayers that are destabilized upon variations in pH, initiating the drug release. Examples are the combination of poly(allylamine hydrochloride) with poly(styrene sulfonate) [307,308] and chitosan with sodium alginate [309,310]. Table 3 summarizes the different pH-responsive approximations described in Section 6.1.

### 6.2. Redox-Responsive MSNs

Unlike healthy cells, where the production of reactive oxygen species (ROS) and antioxidants is balanced, ROS levels in cancer cells are upregulated due to the altered metabolism, some genetic mutations and mitochondrial dysfunction. To counteract this and prevent apoptosis, cancer cells present elevated levels of ROS scavengers, being the most representative the tripeptide glutathione (γ-glutamyl-cysteinyl-glycine, GSH) [311], although the redox-active nature of the endocytic pathway has also been reported [312]. GSH, which participates in the metabolism of many molecules in healthy cells [313], also plays an important role in cancer progression and may contribute to increase radio- and chemoresistance of cancer cells [314]. GSH is mainly located in the mitochondria and cytoplasm, and its upregulated levels (2–10 mM in the cytosol vs. 2–20 µM in the extracellular microenvironment) can be employed to trigger the drug release from redox-responsive mesoporous nanoparticles [315].

Such systems are commonly engineered using different gatekeepers grafted using GSH-cleavable disulfide bonds. In this manner, the pores remain closed outside the cells (bloodstream and tumoral matrix), whereas the overexpressed GSH triggers the release once in the cytoplasm. Examples of gatekeepers attached using disulfide bonds are: (1) proteins, (2) small nanoparticles and nanovalves and (3) polymers and small molecules. In addition to being useful as cleavable linkers for grafting gatekeepers, disulfide bonds can be introduced throughout the framework of PMONs, yielding biodegradable silica nanoparticles in which drug release and degradation are triggered by the presence of overexpressed reductive species [243,316,317].

#### 6.2.1. Proteins

Owing to their large size, proteins are effective in blocking the mesopores when grafted viaredox-responsive bonds. For instance, biocompatible bovine serum albumin (BSA) can be doped with Gd to act at the same time as gatekeeper and magnetic resonance imaging agent [318]. Similarly, transferrin can be employed as both gatekeeper and targeting agent [142]. Cytochrome c, which is an apoptotic protease, can be employed as gatekeeper as well, leading to enhanced doxorubicin delivery and protein-mediated therapeutic effect [319].

#### 6.2.2. Small Nanoparticles and Nanovalves

The pore entrances of MSNs can additionally be sealed by grafting small nanoparticles (4–6 nm) to the surface via disulfide bond. Examples of this approach are the use of carbon dots [320,321], gold nanoparticles [322], silver nanoparticles [323] and cerium oxide nanoparticles [324] as gatekeepers.

The stalks that interact with supramolecular nanovalves can be grafted to the surface of the particles through disulfide bonds, yielding pH- and redox-responsive MSNs. In this manner, enhanced drug release can be observed when MSNs reach the cytoplasm and GSH completely removes the β-CD caps [325,326]. A nice feature of β-CDs is that not only they serve as gatekeeper, but also provide possibilities for further functionalization, including PEGylation [327] and grafting of targeting agents [149,201,328,329].

#### 6.2.3. Polymers and Small Molecules

Disulfide bonds can also be employed to graft bulky polymers to the surface of MSNs. In this regard, cationic polymers such as chitosan [330] and PEI [209,331] have been grafted through redox-responsive bonds to engineer gatekeepers that also serve as gene transfection vectors. Additional examples of gatekeepers grafted through disulfide bonds are naturally occurring polymers, such as HA [158,332] and collagen [152], and synthetic polymers, such as poly(acrylic acid) [333] and poly(glycicyl methacrylate) [334].

Similarly, the pore entrances can be sealed using small disulfide-bridged molecules [335]. Besides, grafting peptides containing hydrophobic / bulky components and targeting units through disulfide bonds provides on-demand drug release and selective recognition of cancer cells [135,196,336,337]. An additional strategy involves grafting stearic acid molecules using GSH-sensitive bonds because such molecules can directly close the mesopores through hydrophobic interactions among them [338]. Moreover, stearic acid can be employed to attach an amphiphilic targeting peptide thanks to their hydrophobic nature, providing both gatekeeping and targeting features [339].

### 6.3. Enzyme-Responsive MSNs

A characteristic feature of tumor environments is the overexpression of certain enzymes with proteolytic behavior. For that reason, designing mesoporous nanomatrices functionalized with gatekeepers degradable by such enzymes has attracted much attention. These materials would avoid premature release in the bloodstream, whereas the payload would be release once in the tumor microenvironment upon enzymatic degradation of the pore blockers. The most targeted enzymes are: (1) cathepsin b (CatB) and (2) various metalloproteinases (MMPs). Besides, (3) the use of some other proteolytic enzymes has also been explored.

#### 6.3.1. Cathepsin B

CatB is a lysosomal proteolytic enzyme which is overexpressed in many cancer cells [340]. In consequence, it can be employed to trigger the drug release from MSNs once they have been internalized through the endocytic pathway. In this sense, MSNs can be capped using large peptides containing repeating units of a CatB-sensitive sequence (GIVRAK) [341]. Similarly, a short CatB-sensitive peptidic sequence (PGFK) can be employed to mask a cationic cell penetrating peptide with a negative complementary chain. In this manner, such interacting chains close the pore entrances until CatB cleaves the sensitive bond along with the negative chain, exposing the positively peptide and targeting the cell nucleus [342]. In addition, such CatB-responsive sequences can be employed for attaching bulky gatekeepers to the surface of MSNs. Examples of such linking sequences are GFLG for attaching α-CDs [343] and CRRGGKKGGKKRK for grafting gold nanoclusters [344]. Besides, MSNs can be coated with poly(glutamic acid), which is able to prevent drug release unless the polymeric coating is degraded by lysosomal CatB [345].

#### 6.3.2. Metalloproteinases

Some MMPs are overexpressed in the tumoral matrix of certain tumors and can be employed to cleave specific peptidic sequences. For instance, the use of peptides containing the MMP-2-responsive PLGVR sequence as gatekeepers for MSNs results in significant drug release only the presence of that enzyme [346,347]. In addition, such responsive sequences can be employed to graft gold nanoparticles and inhibit premature drug release [348]. Similarly, a MMP-9-responsive sequence (RSWMGLP) can be employed to attach avidin to the surface of MSNs, allowing the drug release only in the tumoral matrix [349]. Besides, MSNs can be coated with a MMP-9-sensitive gelatin, so that drug release would only take place upon enzymatic degradation of the polymeric layer [350]. Finally, the pore entrance of MSNs can also be sealed by grafting BSA using a MMP-13-responsive sequence (PLGLAR), because this enzyme has been found to be overexpressed in the tumor microenvironment of liver cancer [351].

#### 6.3.3. Other Enzymes

Aside from serving as targeting agents for the CD44 receptor, HA [156,158,352] and chondroitin sulfate [160,162] can be employed as gatekeepers for enzyme-responsive MSNs, as they are both degraded by hyaluronidase, an enzyme that can be found in the lysosomes. Trypsin is an enzyme that has been found to be overexpressed in liver cancer scenarios. In this respect, it has been shown that the presence of trypsin can degrade BSA in BSA-coated MSNs, triggering the co-delivery of doxorubicin and bilirubin [353].

Similarly, alkaline phosphatase is found in tumor scenarios and can be employed to degrade an ATP coating covering the pores of mesoporous silica-based materials [354]. Finally, cancer cells present elevated levels of esterases in the cytosol, which can hydrolyze ester-containing gatekeepers. Examples of such esterase-responsive systems are the functionalization of MSNs with a poly(β-amino-ester) polymeric layer [355] and ester-containing stalks for supramolecular nanovalves [356,357]. Table 4 summarizes the different strategies applied for the design of enzyme-responsive MSNs.

### 6.4. Light-Responsive MSNs

The use of light to unlock the pore entrances on MSNs has attracted much interest because of the ease of it application, since only a specific light source is needed. Researchers have focused on the use of ultraviolet (UV) (100–400 nm), visible (400–650 nm) and near-infrared (NIR) (650–1050 nm) light. The energy and, consequently, the capacity to penetrate in tissues depends on the wavelength employed. UV light presents the highest energy yet the lowest penetration capacity, which can lead to radiation-induced cellular damage. Conversely, low energetic NIR-light exhibits the deepest penetration capacity in living tissues, in addition to being harmless to the cells [358]. Overall, the design of light-responsive MSNs relies on the use of: (1) breakable bonds upon light irradiation and (2) conformational changes in molecules.

#### 6.4.1. Light-Induced Cleavable Bonds

This kind of bonds can be employed as linkers to graft different gatekeepers to the surface of MSNs. In this manner, the drug release would only take place when the target cells were irradiated by the clinician, assuring lack of non-specific release to healthy cells. For instance, the o-nitrobenzyl group is cleaved upon 365 nm UV-light irradiation and can be employed to close the pore entrances with a targeting protein [140]. In addition, the pH-responsive polymer poly(2-(diethylamino)-ethyl methacrylate) has also be grafted using this linker, endowing MSNs with both pH- and light-responsiveness [359]. Similarly, cationic poly((2-dimethylamino)ethyl methacrylate)) has been anchored to MSNs through a 405 nm-sensitive coumarin group to achieve on-demand gene delivery [360]. Besides, it has been shown that ruthenium bipyridine-based compounds can act as gatekeepers by forming a thiolated coordination bond that can be cleaved upon 455 nm light irradiation [361]. Similarly, the mesopores can be capped with thymine derivatives that undergo reversible formation and cleavage of a cyclobutane dimer when irradiated with 240 nm and 365 nm light to open and close the pores, respectively [362].

As stated in Section 4, photosensitizers are compounds that generate ROS when irradiated with a particular wavelength. In this sense, it is possible to engineer gatekeepers grafted through ROS-responsive bonds that would only allow the release upon application of a given wavelength. For instance, our group has reported the use of a visible light-responsive porphyrin acting as gatekeeper through an aminoacrylate ROS-responsive bond. In this manner, the light irradiation would generate ROS that would then detach the gatekeeper, allowing the drug release [228]. In addition to porphyrins, the photosensitizer chlorin e6 generates ROS upon NIR-light irradiation (660 or 980 nm), and can be loaded in MSNs to mediate the cleavage of ROS-responsive bonds. For instance, β-CD [363] and BSA [364] can be attached using (alkylthio)alkene-based bonds. In addition, it is also possible to use small ROS-responsive thioketal-containing molecules as gatekeepers for MSNs [365]. Finally, the photosensitizer AsPCs_2a_ can be employed to generate ROS able to oxidize the double bonds of the lipid bilayer of lipid-coated MSNs, modifying the permeability and triggering the release [229].

#### 6.4.2. Light-Induced Conformational Changes

Another strategy for the design of light-responsive nanocarriers consists in functionalizing the surface of MSNs with polymers that undergo conformational changes in response to light. This behavior can be accomplished by introducing photoresponsive moieties throughout the polymer chain. In this manner, the light-mediated cleavage of such groups would induce a change from the hydrophobic to hydrophilic state, opening the pores and triggering the drug release. Examples of these groups are perylene (cleaved using 450 nm visible light) [366] and spiropyran (cleaved upon 365 nm UV-light exposure) [367]. A similar approach involves lowering the lower critical solution temperature (LCST) of a thermo-responsive polymer by introducing the o-nitrobenzyl group. In this manner, the bond cleavage upon UV light would increase the LCST of the polymer that would undergo a collapsed to extended conformational change, triggering drug release [368].

Some molecules undergo reversible *trans*-to-*cis* conformational changes upon light irradiation, behavior that many researchers have taken advantage of to engineer light-responsive nanovalves. For instance, using a cinnamamide derivative as stalk for cucurbit[7]uril allows to open and close the mesopores when irradiated with 300 nm and 254 UV-light, respectively [369]. Azobenzene derivatives show similar behavior, and can be employed as stalks for α- [370] and β-CDs [371] that only allow the drug release upon 365 nm UV-light irradiation. Similarly, azobenzene groups can be introduced as pendant groups throughout a polymer chain. In this manner, such groups can interact with the hydrophobic cavities of β-CD-coated MSNs, closing the pores. However, they undergo *trans*-to-*cis* transition when irradiated with 365 nm [372] or 520 nm light [373], ceasing the interactions and allowing the drug release. A nice approach to trigger the drug release consists in grafting fan-like azobenzene derivatives within the mesopores. In this manner, the *trans*-to-*cis* transition acts as a nanoimpeller that forces the payload diffusion outside the pores [374,375]. Table 5 summarizes the different strategies applied for the design of light-responsive MSNs.

### 6.5. Ultrasound-Responsive MSNs

US have long been applied in the clinic for the diagnosis and treatment of different pathologies. The main advantages of US are that their application requires minimal equipment and their non-invasiveness nature that allow deep and harmless penetration in living tissues. For that reason, there has been growing interest in developing US-triggered MSNs that rely on: (1) enhanced drug release owing to effects produced by US and (2) use bonds that can be disrupted by the action of this stimulus.

#### 6.5.1. US-Enhanced Drug Release

Overall, the application of US leads to two main effects, namely acoustic cavitation (as noted in Section 2) and thermal effects, and it has been shown that both effects can lead to significantly higher drug release. In this regard, US can be effectively used to further enhance the effectiveness of functionalized nanoparticles, such as poly(dimethylsiloxane)-coated MSNs [376], polydopamine-coated MSNs [377] and β-CD-coated MSNs [378], achieving more amount of drugs released when used in combination with US.

#### 6.5.2. US-Cleavable Bonds

US can also be employed to weaken chemical interactions, leading to on-off systems upon alternating application of US. Examples of this approach include alginate-coated MSNs that can be cross-linked through the formation of coordination bonds between alginate and calcium ions (COO^—^Ca^2+^) [379] and crown ether-modified MSNs that allow the drug release when hydrogen bonding with the gatekeeper decreases upon US application [380].

Another approximation consists in employing US-cleavable bonds. In this sense, it has been reported that PEG can be mechanically detached from MSNs after applying the stimulus [381]. Our group has reported the use of large co-polymers to seal the pore entrances of the particles. Such polymeric layer contains an US-cleavable acetal, so that when the stimulus is applied the bond is cleaved, leading to a phase transition from hydrophobic to hydrophilic that triggers the drug release [382]. In addition, the versatility of the system can be increased by modifying the synthetic procedure, as that allows the introduction of tunable targeting moieties [129].

### 6.6. Thermo-Responsive MSNs

The variation in temperature required for triggering the drug release from thermo-responsive MSNs can be macroscopically applied or induced by a secondary source, such as an alternating magnetic field or NIR light. The synthesis of this class of materials relies on using (1) gatekeepers that disassemble when there is an increment of temperature and (2) polymers that undergo phase transitions upon temperature variations. This stimulus is particularly interesting in some cases because treating cancer cells with moderate heat (40–43 °C), also known as hyperthermia, results in enhanced cell death and chemosensitivity [383]

#### 6.6.1. Thermo-Responsive Disassembling Gatekeepers

A first strategy to endow MSNs with thermosensitive behavior consists in functionalizing the pore entrances with DNA strands that undergo reversible dehybridization at a given temperature. It would be desirable that the MSNs increased the temperature locally by themselves, so external macroscopic heating would be unnecessary and no surrounding tissues would be affected. In this regard, the DNA nanogates can be grafted to MSNs containing superparamagnetic Fe_3_O_4_ nanoparticles able to generate heat upon application of an alternating magnetic field [384]. Similarly, indocyanine green-loaded MSNs generate heat upon NIR light irradiation, which can be employed to dehybridize DNA strands [231] or complementary base pairs [385]. It has been shown that DNA strands are unable to close the pore entrances below a certain size [386]. Nonetheless, such short sequences can be employed as linkers to block the mesopores with proteins [387], small gold nanoparticles [388] and magnetic γ- Fe_2_O_3_ nanoparticles [389].

In addition to nucleic acids, there are also some examples of thermo-responsive peptidic gatekeepers. For instance, the sequence Phe-Phe-Gly-Gly can be employed to seal the mesopores of MSNs, as it self-assembles at physiological temperature and undergo disassembly when heat is applied. Examples of this strategy include the use of superparamagnetic manganese- and cobalt-doped iron oxide nanoparticles [390] or microwaves [391] to elevate the temperature and trigger drug release. Similar behavior can be accomplished using poly(γ-benzyl-L-glutamate) as pore blocker [392]. Another example is based on using the heterodimeric peptide E/K, because it closes the pore entrances at physiological temperature owing to the coiled coil conformation, which is lost at higher temperature, triggering drug release [393].

MSNs can also be functionalized with thermo-responsive supramolecular nanovalves. For instance, the pores can be sealed using cucurbit[6]uril. Then, the heat generated upon application of a magnetic field is able to disrupt the stalk-nanovalve interaction, triggering the release [394]. Similarly, β-CDs can be attached to the surface using a Diels-Alder-based stalk, which is able to thermally open and close the pores on-demand upon application of the magnetic field [395].

#### 6.6.2. Thermo-Responsive Polymers

There are several examples of polymers that undergo phase transition below and above their LCST. Polymers with a LCST > 37 °C are hydrophobic above physiological temperature. Then, if attached to the surface adopting a hairy conformation, the polymers will be hydrophobic above 37 °C, closing the pores. However, when the polymer is cross-linked and the system is above the transition temperature, that hydrophobic state leaves free spaces in the polymeric network that allow drug release [396]. Among this type of polymers, poly(N-isopropylacrylamide) (pNIPAM) is the most employed. Additional examples are MSNs functionalized with poly(urethane-amine) (LCST ca. 50 °C) [397] or p(MEO_2_MA-co-OEGMA) (LCST ca. 37 °C) [398].

With regard to p(NIPAM), it can be directly engineered as gatekeeper for MSNs, although its coil-to-globule transition taking place at ca. 32 °C complicates its use in patients [399,400]. The LCST can be tuned by introducing different types of monomers. For instance, copolymerizing NIPAM with 3-(methacryloxypropyl)trimethoxysilane) (MPS) raises the LCST of p(NIPAM-co-MPS) up to 36 °C, which would entail better biological performance [401,402]. Similar behavior can be obtained introducing methacrylic acid (MAA), which increases the LCST of p(NIPAM-co-MAA) up to 44.4 °C, endowing magnetic MSNs with both pH- and thermo-responsiveness [403,404]. Our group recently reported magnetic MSNs functionalized with NIPAM and N-(hydroxymethyl)acrylamide) (NHMA). Thermosensitive p(NIPAM-co-NHMA) presented a LCST of ca. 42 °C, showing thermally controlled release and effective hyperthermia treatment in vivo [405,406]. Similarly, p(NIPAM-co-NHMA) can be engineered as gatekeeper for MSNs containing gold nanorods able to generate heat upon NIR light irradiation [137].

There are also polymers that present upper critical solution temperature (UCST). Polymers with UCST > 37 °C are hydrophobic below physiological temperature. For instance, poly(acrylamide-co-acrylonitrile) shows phase transition at 42 °C and can effectively block the pore entrances of MSNs at physiological temperature [407]. Another example is poly(N-acryloyl glycinamide-co-N-phenylacrylamide) (p(NAGAm-co-NPhAm)). This polymer undergoes phase transition at 45 °C and can be grafted to MSNs to accomplish effective drug delivery when the particles generate heat upon NIR-light irradiation [408]. Table 6 summarizes the different strategies applied for the design of thermo-responsive MSNs.

## 7. Future Perspectives

In the last decades, many researchers have taken advantage of the great physico-chemical and biocompatible features of MSNs to design many different nanocarriers for the treatment of different diseases, especially cancer. However, even though there are thousands of publications, the bench-to-bedside translation remains the bottleneck of the field.

Related to the role of industry, an important issue that should be addressed concerns the scalability and reproducibility of the synthesis of the particles. MSNs should be able to be produced on a large scale while showing size and colloidal stability reproducibility. Otherwise, the results derived from potential clinical studies would not have scientific relevance and consistency and health agencies would probably reject such mesoporous silica nanodevices. In this regard, the drug loading in the particles should be completely standardized, as it would be unacceptable that the amount of drug administered varied from one batch to another.

Assuming that the particles could be properly produced, a mandatory step should be the evaluation of these materials in humans, as no realistic translation might be accomplished until assuring the intrinsic toxicity that MSNs might have. Nonetheless, we should be encouraged by the fact that: (a) amorphous silica is “generally recognized as safe” by the FDA and (b) amorphous silica c-dots have been proved to be well tolerated and to accumulate in tumors in human trials. In view of that, both academia and industry should aim to validate in humans the remarkable preclinical results of these cost-effective silica materials.

Once the large-scale production and human safety of MSNs is validated, the next steps should be headed toward implementing the strategies described in this manuscript for overcoming the different biological barriers. In particular, researchers should mainly focus on achieving significant accumulation of nanoparticles in the tumors as, otherwise, they might not constitute a reliable alternative to the systemic administration of free drugs. More importantly, we should bear in mind that nanoparticle-based cancer treatments are not a magic bullet, meaning that they might not be applicable to every single type of cancer. Hence, the physiology of all types of cancer should be completely understood in order to decide whether it is worth treating them with nanomedicine.

In summary, the proper clinical translation of MSNs will require meeting, at least, the previous objectives along with the collaborative and interdisciplinary efforts of scientists and industry.

## 8. Conclusions

Experimental and translational research should focus on the different biological barriers that nanoparticles have to face upon administration, as they currently constitute a bottleneck that is preventing many nanomedicines for achieving effective translation into the clinic. In this sense, the great features that mesoporous silica nanoparticles offer have boosted their application for cancer treatment. It has been shown throughout this review that mesoporous silica nanoparticles can be conveniently engineered to enhance the accumulation in tumor tissues, improve the uptake by tumoral cells and prevent endosomal sequestration. In addition to effectively overcoming such biological barriers, mesoporous silica nanoparticles can be functionalized with many different stimuli-responsive gatekeepers, endowing them with on-demand and localized drug delivery to cancer cells. Furthermore, MSNs all well tolerated in vivo and it has been shown that they are mainly excreted through the urine. In view of the evidences, even more emphasis should be place on mesoporous silica nanoparticles research, as they are attractive candidates for clinic translation in the near future.

## Figures and Tables

**Table 1:** Summary of all the targeting agents implemented into MSNs.

Targeting Agent	Membrane Receptor	Cell Line	Reference
**Antibodies**			
Trastuzumab	HER2	SK-BR3, BT-474	[96,97,98,99,100]
Anti-CD44	CD44	MCF-7	[101]
TRC105	CD105	4T1	[102,103,104]
Anti-EpCAM	EpCAM	Y79	[105]
TAB-004	MUC1	MMT	[106]
Cetuximab	EGFR	MCF-7, PC9, AsPC-1, PANC-1, MIA PaCa-2	[107,108,109]
**Aptamers**			
EpCAM	EpCAM	Huh-7, HepG2, SW620, SW480	[111,112,113,114]
MUC1	MUC1	MDA-MB-231, MCF-7	[115,116]
AS1411	NCL	HeLa, SKOV-3, MCF-7	[117,118,119,120,121]
YQ26	END	HEK293	[122]
HB5	HER2	SK-BR-3	[123]
**Peptides**			
TAT	Importin α/β	HeLa	[126]
KALA	-	A549, HeLa	[127]
RGD	α_v_β_3_-integrin	MDA-MB-231, HeLa, UMR-106, PC-3, 4T1, HUVEC	[128,129,130,131,132,133,181]
NGR	CD13	C6, NCI-H1299, BCEC	[134,135]
NAPamide	Melanocortin-1	#17 (melanoma cancer cells)	[137]
Bld-1	FPR-1	HT-1376	[138]
IL-13	IL-13R-α2	U87	[139]
**Proteins**			
Transferrin	TfR	HT1080, HepG2, Huh-7, MDA-MB-231, C6, MIA PaCa-2	[140,141,142,143,144,145]
Urokinase plasminogen activator	UPAR	S2-VP10	[146]
Concanavalin A	Sialic acids	HOS	[147]
Aleuria Auranti	Sialyl-Lewis X antigen	DLD-1	[148]
**Carbohydrates**			
Galactose	ASGPR	HepG2, SMMC-7721	[149]
Lactobionic acid			[150,151,152,153]
Glucose derivatives	GLUT	Y79, HeLa, A549	[154,155]
Hyaluronic acid	CD44	MDA-MB-231, HCT-116, HeLa, MCF-7	[156,157,158,159]
Chondroitin sulfate			[160,161,162]
**Small Molecules**			
Folic acid	FR-α	PANC-1, LS174T, LnCAP, KB, HeLa, Y79, A549, NCI-H1299	[164,165,166,167,168,169,170,171,172]
Biotin	BR	A549, HeLa, NB-4	[173,174,175]
Boronic acid	Sialic acids	HepG2	[178]
Benzylguanidine derivatives	NET	NB-1691	[180]

**Membrane receptors**. **HER2** (Human epidermal growth factor receptor 2); **CD44** (Cluster of differentiation 44, glycoprotein); **CD105**/**END** (Endoglin protein); **EpCAM** (Epithelial cell adhesion molecule); **MUC1** (Mucin 1 protein); **EGFR** (Epidermal growth factor receptor 1); **NCL** (Nucleolin protein); **CD13** (Aminopeptidase N enzyme); **FPR-1** (Formyl peptide receptor 1); **IL-13R-α2** (Interleukin-13 receptor α2); **TfR** (Transferrin receptor); **UPAR** (Urokinase plasminogen activator receptor); **ASGPR** (asialoglycoprotein receptor); **GLUT** (Glucose transporter); **FR-α** (Folic acid receptor); **BR** (Biotin receptor); **NET** (Norepinephrine transporter). **Cell lines**. **Breast**. SK-BR3 (adenocarcinoma); BT-474 (ductal carcinoma); MCF-7 (invasive ductal carcinoma); MDA-MB-231 (adenocarcinoma); 4T1 (mouse breast cancer that simulates stage IV human breast cancer); MMT (mouse breast cancer). **Lung**. PC9 (adenocarcinoma); A549 (adenocarcinoma); NCI-H1299 (large cell carcinoma). **Pancreas**. AsPC-1 (ductal adenocarcinoma); PANC-1 (ductal carcinoma); MIA PaCa-2 (ductal carcinoma); S2VP10 (ductal adenocarcinoma). **Colon**. SW620 (adenocarcinoma); SW480 (adenocarcinoma); DLD-1 (adenocarcinoma); HCT-116 (carcinoma); LS174T (adenocarcinoma). **Liver**. Huh-7 (hepatocellular carcinoma); HepG2 (hepatoblastoma). **Ovary**. SKOV-3 (ovarian serous cystadenocarcinoma). **Prostate**. PC-3 (carcinoma); LnCAP (carcinoma). **Endocervix**. HeLa (papillomavirus-related endocervical adenocarcinoma); SMMC-7721 (papillomavirus-related endocervical adenocarcinoma); KB (papillomavirus-related endocervical adenocarcinoma). **Bone**. HT-1080 (fibrosarcoma); HOS (osteosarcoma); NB-4 (acute promyelocytic leukemia); UMR-106 (rat osteosarcoma). **Brain**. NB-1691 (neuroblastoma); BCEC (brain capillary endothelial cells); C6 (rat malignant glioma). **Eyes**. Y79 (retinoblastoma). **Kidney**. HEK293 (embryonic human kidney cells). **Bladder**. HT-1376 (carcinoma). **Endothelium**. HUVEC (human umbilical vein endothelial cells).

**Table 2:** Summary of the different strategies implemented into MSNs for achieving dual or hierarchical targeting.

Targeting Agents	Approach	Cell line	Reference
**Dual Targeting**			
Hyaluronic acid + RGD	Two different membrane targeting agents	SKOV-3	[181,182]
Biotin + Folic acid		HeLa	[183]
Bevacizumab + EpCAM aptamer		SW480	[184]
Benzylguanidine derivatives	Y-shaped scaffold using the same membrane targeting agent	NB-1691	[185]
RGD + TAT	Sequential vascular-membrane-organelle targeting	HeLa	[186]
Folic acid + Dexamethasone	Sequential membrane-organelle targeting	HeLa	[187]
Hyaluronic acid + Triphenylphosphonium		MGC-803	[188]
Hyaluronic acid + Positive charge	Janus dual membrane targeting	A549	[189]
Folic acid + TPP	Janus membrane-organelle targeting	LnCAP	[190]
**Hierarchical Targeting**			
Positive charge	pH-responsive benzoic imine bond	HepG2, HeLa	[193,194,195]
RGD		U87	[196]
Positive charge	Thermally-cleavable bond	HOS	[197]
Hyaluronic acid	MMP-2-degradable gelatin	MDA-MB-231	[198]
Folic acid		HT-29	[199]
RGD	RGD masked with MMP-2-clevable peptide sequence	4T1, HT-29	[200,201]

**Cell lines**. **Breast**. MDA-MB-231 (adenocarcinoma); 4T1 (mouse breast cancer that simulates stage IV human breast cancer); MMT (mouse breast cancer). **Lung**. A549 (adenocarcinoma). **Colon**. SW480 (adenocarcinoma); HT-29 (adenocarcinoma). **Liver**. HepG2 (hepatoblastoma). **Ovary**. SKOV-3 (ovarian serous cystadenocarcinoma). **Prostate**. LnCAP (carcinoma). **Endocervix**. HeLa (papillomavirus-related endocervical adenocarcinoma); **Bone**. HOS (osteosarcoma). **Brain**. NB-1691 (neuroblastoma); U87 (glioblastoma). **Stomach**. MGC-803 (adenocarcinoma).

**Table 3:** Different strategies implemented into MSNs for achieving pH-responsive drug delivery.

Approach	Description	Reference
**Acid-Labile Bonds**		
Hydrazone bond	pH-responsive bonds that find application as linkers between MSNs and different gatekeepers	[248,249,251]
Acetal bond		[147,252,253,254,255,256]
Boronate ester bond		[258,259,260,261]
Imine bond	pH-responsive bond useful as cross-linking agent	[262,263,264]
**pH-Degradable Gatekeepers**		
Inorganic nanoparticles	Small nanoparticles that degrade at acid pH, generating different ions with therapeutic applications	[265,266,267,268,269,270,271]
Polymers	Polymeric coatings that decompose into their building blocks upon changes in pH	[272,274,275,276]
**pH-Operated Nanovalves**		
Stalk + Cap	Supramolecular structures that close and open the pores thanks to the interaction with stalks grafted on the surface	[277,278,279,280,281]
**Conformation-Changing Polymers**		
Cationic	Polymers that are collapsed on the surface of the particles when deprotonated (pores closed) and undergo a conformational change when pH varies (pores open)	[230,282,283,284,285,286,287,288]
Anionic		[289,290,291]
**pH-Responsive Biomolecules**		
Polypeptides	Large peptidic chains containing protonable groups that exhibit collapsed-to-extended behavior upon pH variations	[215,292,293]
Nucleic acids and proteins	Macromolecules that modify their 3D structure upon variations in pH	[294,295,296,297,298,299]
**Polyelectrolytes**		
Monolayers	Charged polymers that close the pores by forming a monolayer through electrostatic interactions	[300,301,302,303,304,305,306]
Multilayers	Arrangement of multiple charged layers on the surface of the particles to close the pore entrances	[307,308,309,310]

**Table 4:** Strategies implemented into MSNs for achieving enzyme-responsive drug delivery.

Enzyme	Description	Reference
**Peptides as Gatekeepers**		
CatB	Large peptidic sequences that close the mesopores and allow drug release upon enzymatic degradation	[341,345]
MMP-2		[346,347]
**Peptide as Linkers**		
CatB	Short peptidic sequences employed to graft different types of bulky gatekeepers (small nanoparticles, proteins, nanovalves) to the surface of MSNs	[342,343,344]
MMP-2		[348]
MMP-9		[349]
MMP-13		[351]
**Other Enzyme-Degradable Gatekeepers**		
MMP-9	MSNs covered with a gelatin that degrade in the presence of MMP-9	[350]
Hyaluronidase	MSNs functionalized with large carbohydrates (hyaluronic acid, chondroitin sulfate) that act simultaneously targeting agents and gatekeepers	[156,158,160,162,352]
Trypsin	MSNs gated with BSA, which can be degraded by overexpressed trypsin in liver cancer	[353]
Alkaline phosphatase	ATP-capped mesoporous silica-based materials that allow drug release upon enzymatic degradation of ATP	[354]
Esterases	MSNs functionalized with ester-containing gatekeepers that are degraded in the presence of such enzymes	[355,356,357]

**Table 5:** Different strategies implemented into MSNs for achieving light-responsive drug delivery.

Approach	Description	Reference
**Light-Responsive Bonds**		
o-nitrobenzyl group	Cleavable using 365 nm light. Used as linker for the grafting of proteins and pH-responsive polymers	[140,359]
Coumarin group	Cleavable using 405 nm light. Used as linker for the grafting of cationic polymers for gene delivery	[360]
Thiolated coordination bond	Cleavable using 455 nm light. Coordination bond formed by ruthenium bipyridine-based compounds that act as gatekeepers	[361]
Thymine derivatives	Reversible formation (365 nm) and cleavage (240 nm) of a cyclobutane dimer. Used as on-off gatekeeper	[362]
**ROS-Responsive Bonds (ROS Generation upon Light Application)**		
Aminoacrylate bond	Used as linker for the grafting of a porphyrin acting simultaneously as gatekeeper and ROS generator upon visible light irradiation	[228]
(alkylthio)alkene-based bond	Used as linker for the grafting of nanovalves and proteins. Cleaved when loaded photosensitizer chlorin e6 generates ROS upon NIR light irradiation	[363,364]
Thioketal group	Used as gatekeeper. Cleaved when chlorin e6 generates ROS upon NIR light irradiation	[365]
Double bonds	Photosensitizer AsPCs_2a_ generates ROS upon NIR light irradiation that oxidize double bonds of the lipids and increase membrane permeability	[229]
**Light-Induced Conformational Changes**		
Perylene group	Their removal from a polymer chain upon light irradiation, 450 nm (perylene) or 365 nm (spiropyran and o-nitrobenzyl), induce a conformational change that open the pores and triggers drug release	[366]
Spiropyran group		[367]
o-nitrobenzyl group		[368]
Cinnamamide derivative	Used as stalks for grafting of nanovalves (grafted on the surface or as pendant groups in polymers). Reversible *trans*-to-*cis* conformation change when irradiated, in general, with UV light	[369]
Azobenzene derivatives		[370,371,372,373,374,375]

**Table 6:** Different strategies implemented into MSNs for achieving light-responsive drug delivery.

Approach	Description		Reference
**Thermo-Responsive Disassembling Gatekeepers**			
DNA	Large DNA strands acting as gatekeepers that dehybridize above a certain temperature, triggering drug release		[231,384,385,386]
	Short DNA strands used as linkers for grafting bulky gatekeepers (proteins, small nanoparticles) that allow drug release when strands dehybridize upon heating		[387,388,389]
Peptides	Peptide sequences used as gatekeepers that self-assemble at physiological temperature and undergo disassembly when heated		[390,391,392]
	Heterodimeric peptide acting as gatekeeper that present a coiled coil conformation at physiological temperature. That 3D structure is lost when heat is applied, triggering drug release		[393]
Nanovalves	Supramolecular nanovalves attached to the surface through thermo-sensitive stalks		[394,395]
**LCST Polymers (Hydrophobic if T > LCST)**			
poly(urethane-amine)	LCST ca. 50 °C	Polymers form a polymeric network. Below LCTS hydrophilic chains hamper drug release. When T > LCTS, polymers become hydrophobic and the polymeric network shrinks, facilitating drug release.	[397]
p(MEO_2_MA-co-OEGMA)	LCST ca. 37 °C		[398]
p(NIPAM)	LCST ca. 32 °C		[399,400]
p(NIPAM-co-MPS)	LCST *ca*. 36 °C		[401,402]
p(NIPAM-co-MAA)	LCST ca. 44 °C		[403,404]
p(NIPAM-co-NHMA)	LCST ca. 42 °C		[137,405,406]
**UCST Polymers (Hydrophilic if T > UCST)**			
poly(acrylamide-co-acrylonitrile)	UCST ca. 42 °C	Polymers become hydrophilic above UCST, adopting an extended conformation that triggers drug release	[407]
p(NAGAm-co-NPhAm)	UCST ca. 45 °C		[408]
